# Identification of a DNA damage repair-related LncRNA signature for predicting the prognosis and immunotherapy response of hepatocellular carcinoma

**DOI:** 10.1186/s12864-024-10055-1

**Published:** 2024-02-08

**Authors:** Fei Huang, Chunyan Zhang, Wenjing Yang, Yan Zhou, Yihui Yang, Xinrong Yang, Wei Guo, Beili Wang

**Affiliations:** 1 Department of Laboratory Medicine, Zhongshan Hospital, Fudan University, Shanghai, China; 2 Department of Laboratory Medicine, Shanghai Geriatric Medical Centre, Shanghai, China; 3 Department of Laboratory Medicine, Xiamen Branch, Zhongshan Hospital, Fudan University, Xiamen, China; 4 Department of Laboratory Medicine, Wusong Branch, Zhongshan Hospital, Fudan University, Shanghai, China; 5 Department of Liver Surgery & Transplantation, Liver Cancer Institute, Zhongshan Hospital, Fudan University, Shanghai, China

**Keywords:** DNA damage repair, Hepatocellular carcinoma, LncRNA signature, Immune infiltration, Therapeutic response

## Abstract

**Background:**

DNA damage repair (DDR) may affect tumorigenesis and therapeutic response in hepatocellular carcinoma (HCC). Long noncoding RNAs (LncRNAs) can regulate DDR and play a vital role in maintaining genomic stability in cancers. Here, we identified a DDR-related prognostic signature in HCC and explored its potential clinical value.

**Methods:**

Data of HCC samples were obtained from the Cancer Genome Atlas (TCGA), and a list of DDR-related genes was extracted from the Molecular Signatures database (MSigDB). A DDR-related lncRNAs signature associated to overall survival (OS) was constructed using the least absolute shrinkage and selection operator-cox regression, and was further validated by the Kaplan-Meier curve and receiver operating characteristic curve. A nomogram integrating other clinical risk factors was established. Moreover, the relationships between the signature with somatic mutation, immune landscape and drug sensitivity were explored.

**Results:**

The prognostic model of 5 DDR-related lncRNAs was constructed and classified patients into two risk groups at median cut-off. The low-risk group had a better OS, and the signature was an independent prognostic indicator in HCC. A nomogram of the signature combined with TNM stage was constructed. TP53 gene was more frequently mutated in the high-risk group. Marked differences in immune cells were observed, such as CD4 + T cells, NK cells and macrophages, between the two groups. Moreover, an increase in the expression of immune checkpoint molecules was found in the high-risk group. The low-risk group presented with a significantly higher response to sorafenib or cisplatin. Finally, potential value of this signature was validated in real-world HCC patients.

**Conclusion:**

Our findings provided a promising insight into DDR-related lncRNAs in HCC and a personalized prediction tool for prognosis and therapeutic response.

**Supplementary Information:**

The online version contains supplementary material available at 10.1186/s12864-024-10055-1.

## Background

Hepatocellular carcinoma (HCC), the most common type of primary liver cancer, is the fourth leading cause of cancer-related death worldwide [1]. Even with advances in diagnostic approach and therapeutic management, the 5-year survival rates remain low [2]. Thus, more prognostic markers are still urgently needed to predict HCC prognosis and guide personalized therapy for patients.

The occurrence of HCC has been closely associated with various risk factors, such as virus infection, alcohol addiction, metabolic liver disease and exposure to toxins, which may induce DNA damage [3]. DNA damage repair (DDR) mechanisms, such as direct reverse repair, base excision repair, nucleotide excision repair, mismatch repair, and double-strand break (DSB) repair, activated following DNA damage, are responsible for maintaining genomic stability. Long-term dysfunction of the DDR may lead to the activation of hepatocarcinogenesis and further progression [4]. Moreover, the loss of DDR function may determine the response of anticancer treatment, as previously reported [5, 6].

Long non-coding RNAs (lncRNAs) refer to a class of non-coding transcripts with lengths over 200 nucleotides, which take part in gene expression regulation [7]. Previous studies have shown that lncRNAs may be involved in the regulation of the DDR processes [8–10]. Moreover, DDR-related lncRNAs may be involved in HCC progression [11]. LncRNA PRLH1 can bind to the DNA repair protein RNF169, and promote the recruitment and retention of RNF169, thereby promoting homologous recombination repair and increasing proliferation in HCC cells [12]. LINC02163 can regulate the nonhomologous end joining repair pathway by binding to effector proteins promote the ligation efficiency of blunt-ended DSB, thereby maintaining proliferation [13]. Furthermore, lncRNAs are considered as crucial regulators between cancer cells and immune cells in the tumor microenvironment (TME), which can interfere with immune responses to affect the therapeutic efficiency [14]. However, the application of the DDR-related lncRNAs signature in prognostic prediction for HCC patients remains to be elucidated.

In this study, we screened DDR-related lncRNAs with prognostic value from the Cancer Genome Atlas (TCGA) and constructed a risk signature. Furthermore, bioinformatics studies were performed to investigate the correlations between the risk score with immune infiltration and therapeutic response. Finally, we analyzed the expression of these lncRNAs in HCC cell lines and plasma samples and investigated their relations with clinical features of the patients.

## Materials and methods

### Data collection and patients enrollment

Transcriptome sequencing data, somatic mutation data and corresponding clinical information of HCC samples were downloaded from TCGA database (https://portal.gdc.cancer.gov/repository), including 374 tumor samples and 50 normal samples. After excluding the samples with prior malignancy and therapy, a total of 334 tumor samples were included in the further analysis. In addition, 450 DDR-related genes were assembled from the Molecular Signatures database (MSigDB) (https://www.gsea-msigdb.org/gsea/index.jsp), which were listed in Table S1.

Additionally, a total of 50 patients with HCC at Zhongshan Hospital Fudan University between August 2023 and September 2023 were enrolled. Enrollment criteria were as follows: (1) definite HCC diagnosis based on histopathological examinations; and (2) age > 18 years. Exclusion criteria were as follows: (1) having history of any malignancy; (2) having history of the prior treatment; and (3) pregnant woman. In addition, a total of 15 healthy controls (HC) and 25 patients with benign lesions (BL), including hepatic hemangioma, hepatic cyst and focal nodular hyperplasia, were enrolled. Approval for the use of human subjects was obtained from the Research Ethics Committee of Zhongshan Hospital (B2022-435R). Informed consents were obtained from each individuals in this study.

### Identification of DDR-related lncRNAs

The “limma” package in R was applied to normalize and identify differentially expressed genes (DEGs) and lncRNAs (DE-lncRNAs) between tumor and normal samples with the threshold of |log (fold change)| > 1.5 and a false discovery rate (FDR) < 0.05. A total of 76 intersecting genes were identified. DDR-related lncRNAs were identified using Pearson correlation analysis based on the criteria of |r| > 0.5 and *P* < 0.001.

### Construction and validation of the DDR-related lncRNAs risk model

Univariate cox regression was performed to filter out lncRNAs related to overall survival (OS), and those with *P* < 0.05 were considered as potential candidates. Liver hepatocellular carcinoma (LIHC) samples from TCGA were randomly divided into a training group and a testing group at a ratio of 1:1. Then, least absolute shrinkage and selection operator (LASSO)-cox regression based on package “glmnet” in R was used to construct a prognostic risk model. Finally, a five-lncRNAs model was established, multiplied by the coeffificients and the corresponding lncRNAs expression to calculate the risk score of each samples. Samples were categorized as high- or low-risk at the median cut-off. Kaplan-Meier (K-M) curve was utilized to compare OS between the high- and low-risk groups. Receiver operating characteristic (ROC) curve was used to assess the sensitivity and specificity of the model. All analyses were performed in the internal training group, testing group, and all samples.

### Correlations between the risk model and clinical characteristics

Univariate and multivariate cox regression analyses were used to explore independent prognostic features for HCC (including risk score, age, gender, grade and TNM stage). For univariate analysis, features with a *P* < 0.05 were included in multivariate analysis. Based on the multivariate analysis, a nomogram was established using the “rms” package in R to predict the 1-, 3- and 5-year OS for HCC.

### Molecular landscape in different risk groups

We studied the landscape of somatic mutations available in the high- and low-risk groups. Analyses of somatic mutation frequency and tumor mutation burden were conducted by “maftools” package in R.

### Assessment of immune cell infiltration

Six different algorithms were used to compute the relative fraction of immune cell populations, including CIBERSORT, TIMER, MCPCounter, EPIC, quanTIseq, and xCELL. ESTIMATE and xCELL algorithm were adopted to assess the stromal and immune score in tumor samples. Differences of immune cell infiltration between the high- and low-risk groups were compared using unpaired Student’s *t-*test, and correlations between the risk score and immune cells was assessed using Spearman correlation test.

### Estimation of immunotherapy and chemotherapy

We evaluated the correlations between the risk score and the expression of the immune checkpoint genes, such as CD274, CD276, CTLA4, IDO1, LAG3, PDCD1, TIGIT and VSIR, and also applied “oncoPredict” package in R to predict the response to various chemotherapy.

### Cell culture, tissue and plasma samples

The human fetal hepatocyte LO2, as well as HCC cells Huh7, PLC, LM3, MHCC-97 L and MHCC-97 H are all available in our lab. All cell lines were cultured in DMEM (Gibco, Shanghai, China) supplemented with 10% fetal bovine serum (FBS), 100 U/ml of penicillin sodium, and 0.1 mg/ml of streptomycin sulfate in humidified air containing 5% CO_2_ at 37 ℃. The experimental cells were in the logarithmic growth phase. Plasma samples were collected from each subjects before surgery and stored at -80 °C until analysis.

### RNA extraction and quantitative real-time PCR (qRT-PCR)

Total RNA from cell lines and plasma was extracted using TRIzol reagent (Invitrogen), and then converted to cDNA using the GoScript reverse transcription kit (Promega). All operations were carried out by the manufacturer’s instructions. The real-time PCR system (Applied Biosystems) was used for quantification of lncRNAs level, and the reaction conditions were carried out according to the instructions of the SYBR Green Master Mix (Yeasen). The relative expression of candidate lncRNAs were normalized to GAPDH and was calculated using the 2^−∆∆CT^ method. The sequences of all primers analyzed in this study are provided in Table 1.

**Table 1:** The amplification primer sequences

LncRNA	Primer F	Primer R
AC012073.1	GGAGCTTGGGCTCTTAGGTC	TGACGGTGATGGTGTTCCTC
AL031985.3	AAATCCCATACCCCTTTCACC	TTTACTGAGTCCCTTCTGCGTG
AL355574.1	AAGATGGGAAAGGTCGAGGC	CTCAACACAGCCAAAGCCAC
LINC01224	TCCTGAGAGCCCCAGCTATT	TTTACGGTGGACCAGATGGC
SNHG4	GGCTAGAGTACAGTGGCTCG	GCAAATCGCAAGGTCAGG
GAPDH	GTTACCAGGGCTGCCTTCTC	GATGGTGATGGGTTTCCCGT

### Statistical analysis

All statistical analyses were performed using R (4.3.0), SPSS Statistics 26 (IBM, Chicago, IL, USA), and Graphpad Prism 8 (Graphpad Software, America). Appropriate packages, such as “glmnet”, “limma”, “maftools”, “oncoPredict” and “rms”, were applied for several statistical analyses. Differences between two groups were analyzed using unpaired Student’s *t*-test. Univariate and multivariate cox regression analyses were implemented to define the independent prognostic factor for OS. The predictive capacity of the prognostic model for OS was evaluated by performing K-M curve and time-dependent ROC curve analysis. Statistical significance was defined as *P* < 0.05, and all *P* values were two-tailed.

## Results

### Identification of differentially expressed DDR-related lncRNAs in HCC

**Fig. 1:**
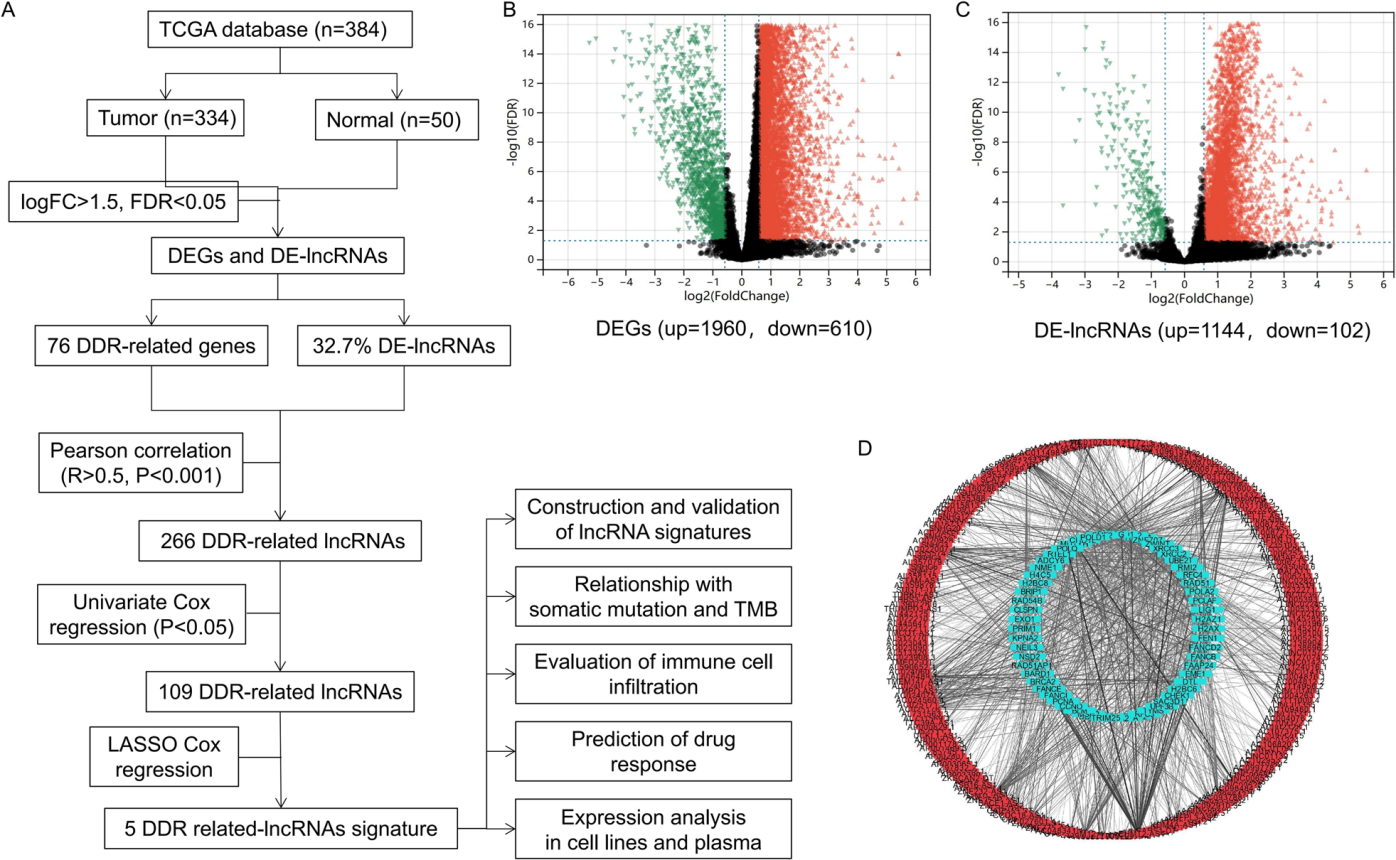
Construction of the DDR-related mRNAs-lncRNAs co-expression network and identifying prognostic DDR-related lncRNAs. **A **The flow chart of this study. Volcano plots of DEGs (**B**) and DE-lncRNAs (**C**) in TCGA-LIHC samples. Red dots represent up-regulated genes and green dots represent down-regulated genes. **D **The coe-xpression network of DDR-related IncRNAs

The workflow of the prognostic model analysis is illustrated in Fig. 1A. We identified 2570 genes and 1246 lnRNA (3104 upregulated and 712 downregulated) differentially expressed in TCGA-LIHC samples (Fig. 1B and C). Through overlapping analysis, 76 DDR-related DEGs were eventually selected out (Table S2). Furthermore, 266 DE-lncRNAs were determined as the DDR-related lncRNAs according to correlation analysis (Fig. 1D). Finally, 109 DDR-related lncRNAs with prognostic value were regarded as potential candidates via univariate cox regression (Table S3).

### Construction of a DDR-related lncRNA prognostic signature

**Fig. 2:**
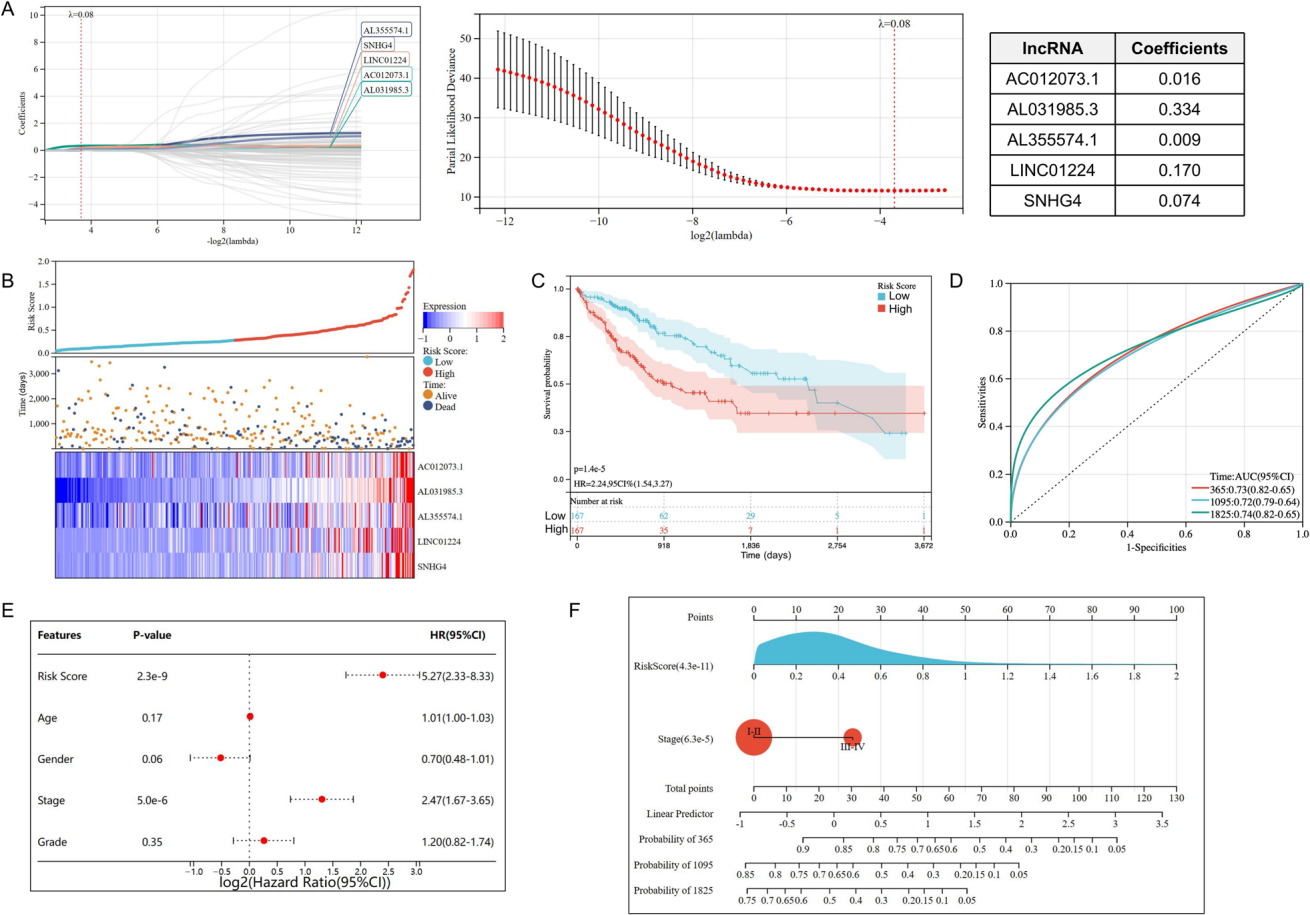
Construction of a DDR-related lncRNAs risk model and the evaluation of independent prognostic potential. **A **Development of a DDR-related lncRNAs risk model using Lasso-Cox regression analysis. **B **The distribution of the risk score and scatter plot of survival in all samples. **C **K-M curve of the high- and low- risk group in all samples. **D **Time-dependent ROC curve for the prognostic prediction of the risk model at 1-, 3- and 5-year survival time in all samples. **E **Univariate cox regression analysis for the risk model as an independent prognostic factor. **F **A nomogram to predict the 1-, 3- and 5-year OS of HCC patients

The risk model based on 5 DDR-related lncRNAs was built by LASSO-cox regression (Fig. 2A). HCC patients were randomly separated into a training group and a testing group. In each group, HCC patients were classified into high- or low-risk groups at median cut-off. More deaths were observed in the high-risk group, and a heatmap revealed the distribution of these lncRNAs in the different risk groups (Fig. 2B, S1A, S1D). All candidate lncRNAs were elevated in the high-risk group. K-M curve indicated that the high-risk group had a poorer OS than the low-risk group in training group, testing group, and overall samples (Fig. 2C, S2B, S2E). In the training group, the area under the curves (AUCs) of risk score for 1-, 3- and 5-year OS were 0.76, 0.71 and 0.73, respectively (Fig. 2D). Similar findings were validated in testing group and all samples (Figure S2C, S1F) to reduce model overfitting. Cox regression analysis was performed to explore whether the lncRNA model was an independent prognostic factor when clinicopathological features (such as age, gender, grade and TNM stage) were included. We found that the risk model [*P* < 0.001, hazard ratio (HR) = 5.27, 95% confidence interval (CI) = 2.33–8.33] and TNM stage were independently related to OS (Fig. 2E). A nomogram containing the lncRNAs signature and TNM stage was established to predict the 1-, 3- and 5-year OS for HCC (Fig. 2F), with the patient’s prognosis worsening as the risk score increased.

### Relationship between risk score and somatic mutation

**Fig. 3:**
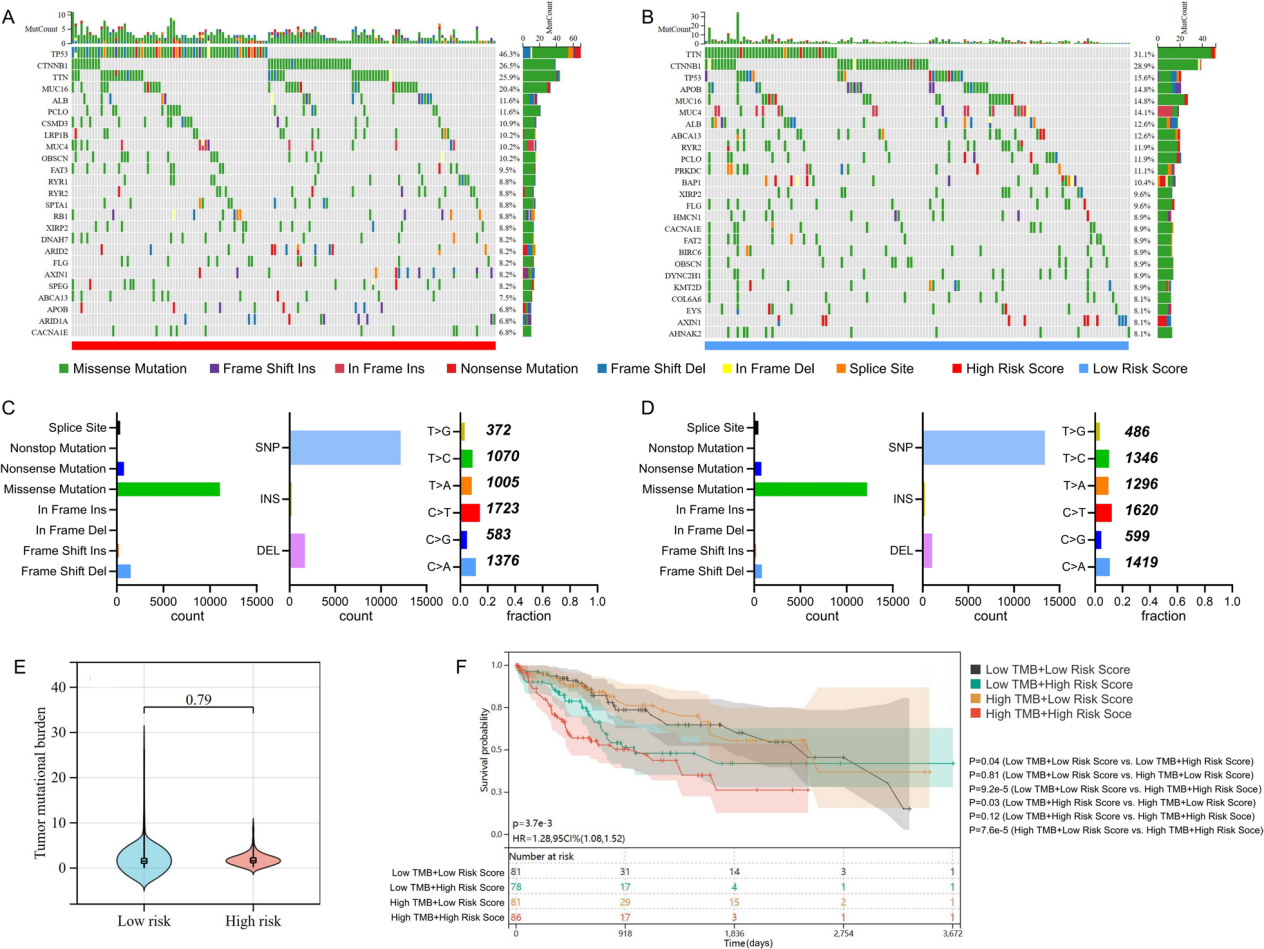
Tumor somatic mutation and differential TMB in two risk groups. The waterfall plot of tumor somatic mutation in samples with high- (**A**) and low-risk score (**B**). Overall somatic alterations in the high- (**C**) and low-risk groups (**D**). **E **Comparison of TMB in two risk groups. **F **Combined survival analysis of TMB and risk score

Oncoplots revealed that the first three mutated genes were TP53, CTNNB1, and TTN, and missense mutations were the most common type of molecular alterations (Fig. 3A, B, C and D). Discrepancies were observed in somatic mutations between the high- and low-risk groups (Fig. 3A and B). Notably, the mutation rates of TP53 was substantially greater in the high- than low-risk group (46.3% vs. 15.6%, *P* < 0.001), suggesting that risk model may be related to the mutation status of TP53. However, no difference was found in tumor mutational burden (TMB) between the two risk groups (Fig. 3E). Between the high- and low- TMB groups, there was no difference in patient survival (*P* = 0.26) (Figure S2). Combining TMB with the risk score, the survival rates of patients in four subgroups were significantly different (*P* < 0.001) (Fig. 3F).

### Landscape immune infiltration in the two risk groups

**Fig. 4:**
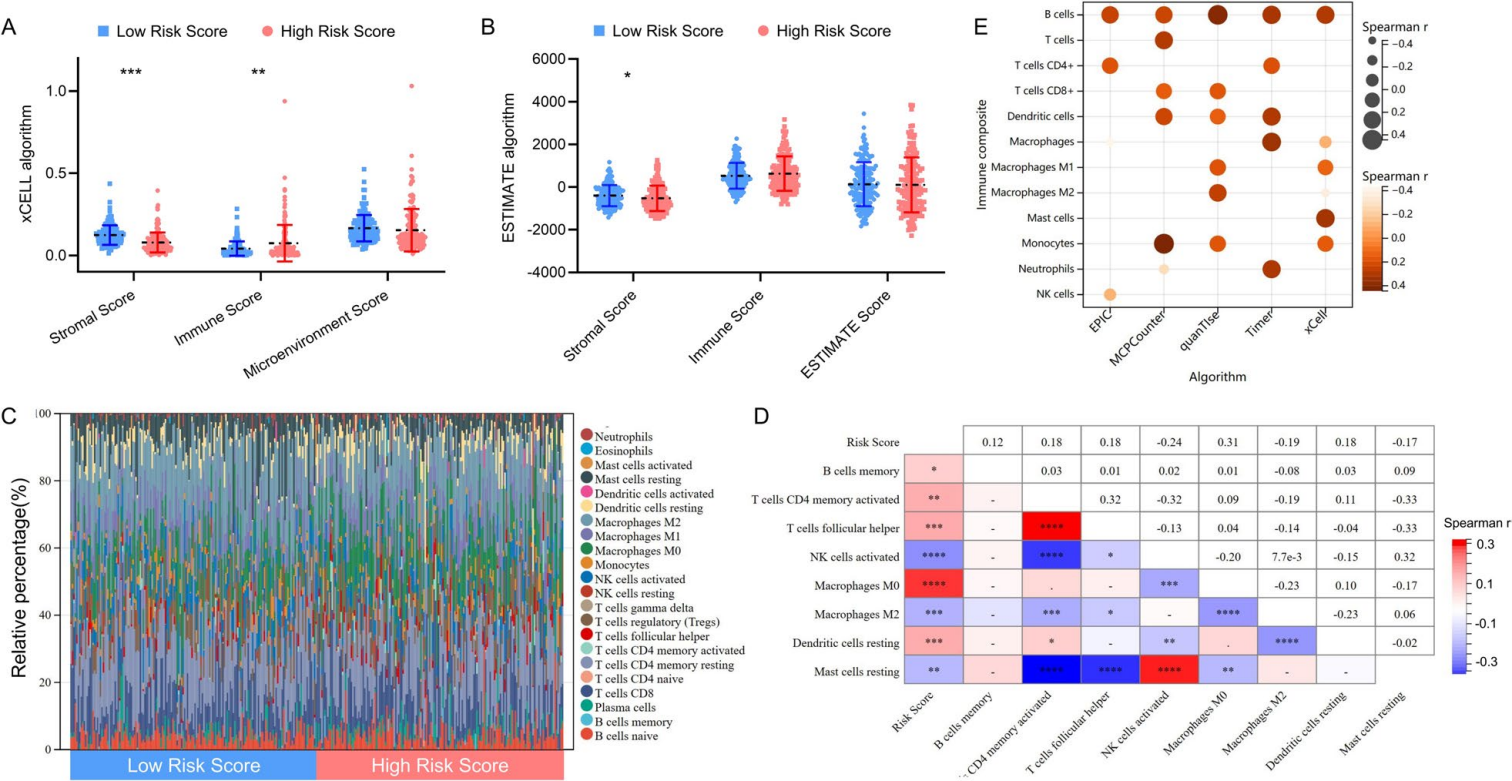
Comprehensive analysis of the DDR-related lncRNAs signature and TME. Differences in TME between high- and low-risk groups based on the xCELL (**A**) and ESTIMATE (**B**) algorithm. **C **The relative percentage of 22 immune cells estimated by CIBERSORT algorithm. **D**-**E **Correlations between risk score and relative immune infiltration score assessed using CIBERSORT, EPIC, MCPCounter, quanTIseq, Timer and xCELL algorithm. ^*^: *P* < 0.05, ^**^: *P* < 0.01, ^***^: *P* < 0.001

TME plays an important role in the development in HCC. We found significant differences in stromal score and immune score between the high- and low-risk groups via the xCell algorithm (Fig. 4A), whereas stromal score was much higher in low-risk group based on the ESTIMATE algorithm (Fig. 4B). CIBERSORT algorithm was used to evaluate immune composite variances between the two risk groups. Patients with high-risk score were found to have a higher proportion of memory B cells, memory-activated CD4 + T cells, follicular helper T cells, M0 macrophages and resting dendritic cells, whereas activated NK cells, M2 macrophages and resting mast cells were relatively lower (Fig. 4C, S3). Additionally, memory-activated CD4 + T cells, follicular helper T cells, M0 macrophages and resting dendritic cells increased as the risk score increased, while activated NK cells, M2 macrophages and resting mast cells decreased (Fig. 4D). Five additional algorithms were used to determine the relationship between risk scores and its immunological components (Fig. 4E). Therefore, these findings implied that the infiltration of these immune cell subtypes might exert an important influence on the prognosis of HCC.

### Evaluation of the immunotherapeutic and chemotherapeutic response

**Fig. 5:**
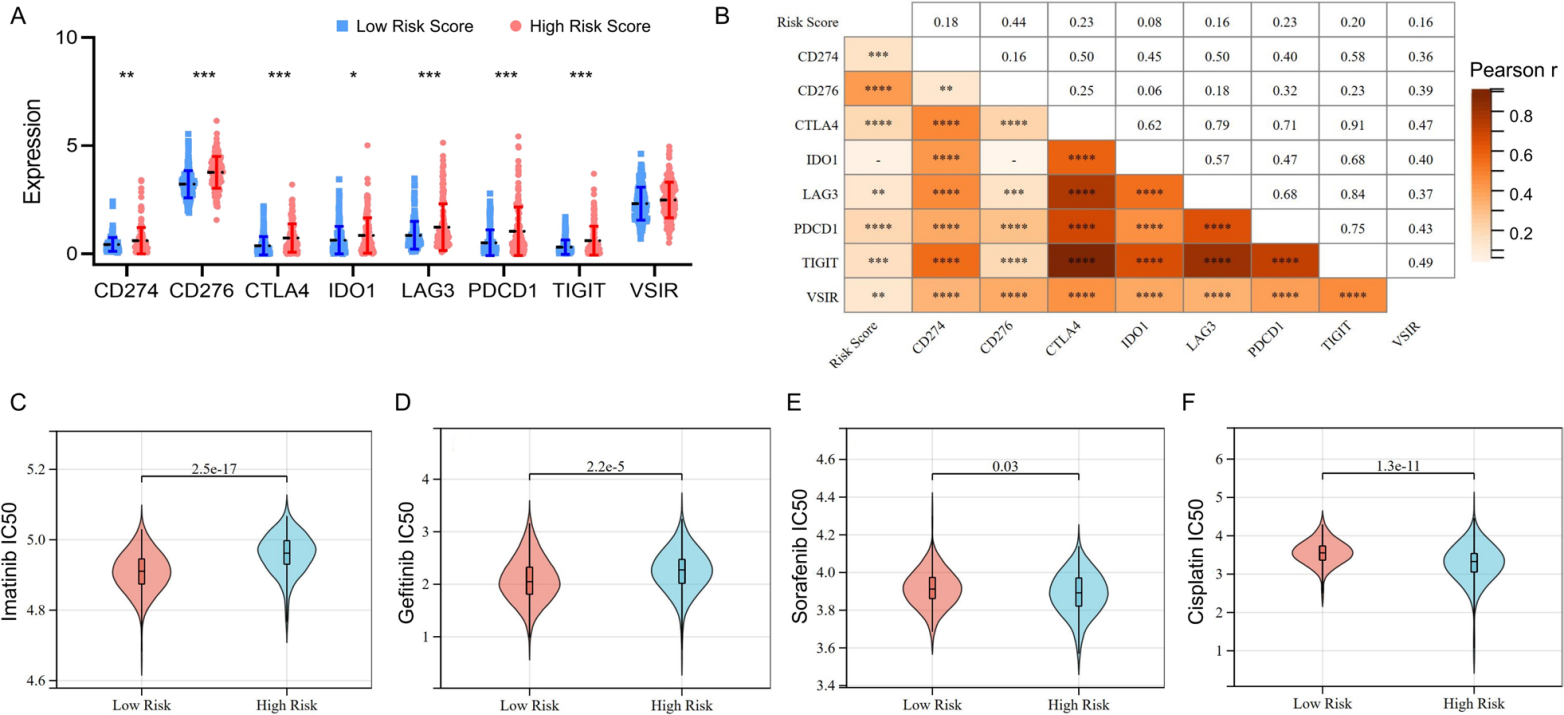
Risk score could predict the clinical benefits of immunotherapy and chemotherapy. **A **Comparison of the immune checkpoints genes between the high- and low-risk groups. **B **Correlation analyses of risk scores with immune checkpoint targets. Differences of the estimated IC50 of imatinib (**C**), Gefitinib (**D**), Sorafenib (**E**) and Cisplatin (**F**) between two risk groups in database. ^*^: *P* < 0.05, ^**^: *P* < 0.01, ^***^: *P* < 0.001

Given the clinical importance of therapeutic strategies based on immune checkpoint blockade in HCC, we explored the association between the risk score and several immune checkpoints. Compared with the low-risk group, expression levels of CD274, CD276, CTLA4, IDO1, LAG3, PDCD1 and TIGIT were significantly higher in the high-risk group (Fig. 5A). Meanwhile, expression levels of CD274, CD276, CTLA4, LAG3, PDCD1, TIGIT and VSIR were positively related to the risk scores (Fig. 5B).

We further proceeded to investigate the potential role of risk score in the setting of chemotherapy. Patients with low-risk score had lower estimated half maximal inhibitory concentration (IC50) values for imatinib (Fig. 5C) and gefitinib (Fig. 5D), indicating that low-risk HCC patients were more resistant to imatinib or gefitinib treatment. Patients with high-risk score had lower estimated IC50 values for sorafenib (Fig. 5E) and cisplatin (Fig. 5F), indicating that high-risk HCC patients were more resistant to sorafenib or cisplatin treatment. Taken together, these data suggested that the DDR-related lncRNAs signature might affect the sensitivity of chemotherapy and immunotherapy in HCC patients.

### Validation of the DDR-related lncRNAs by qRT-PCR

Relative levels of the DDR-related lncRNAs in both cell lines and plasma were detected by qRT-PCR. The expression of AL355574.1 and SNHG4 was decreased in all HCC cell lines. AL031985.3 and LINC01224 was significantly increased in Huh7 and PLC cells but marginally decreased in LM3, MHCC-97 L and MHCC-97 H cells. AC012073.1 was upregulated in PLC, LM3 and MHCC-97 L cells, while it was downregulated in Huh7 cells (Figure S4A).

**Fig. 6:**
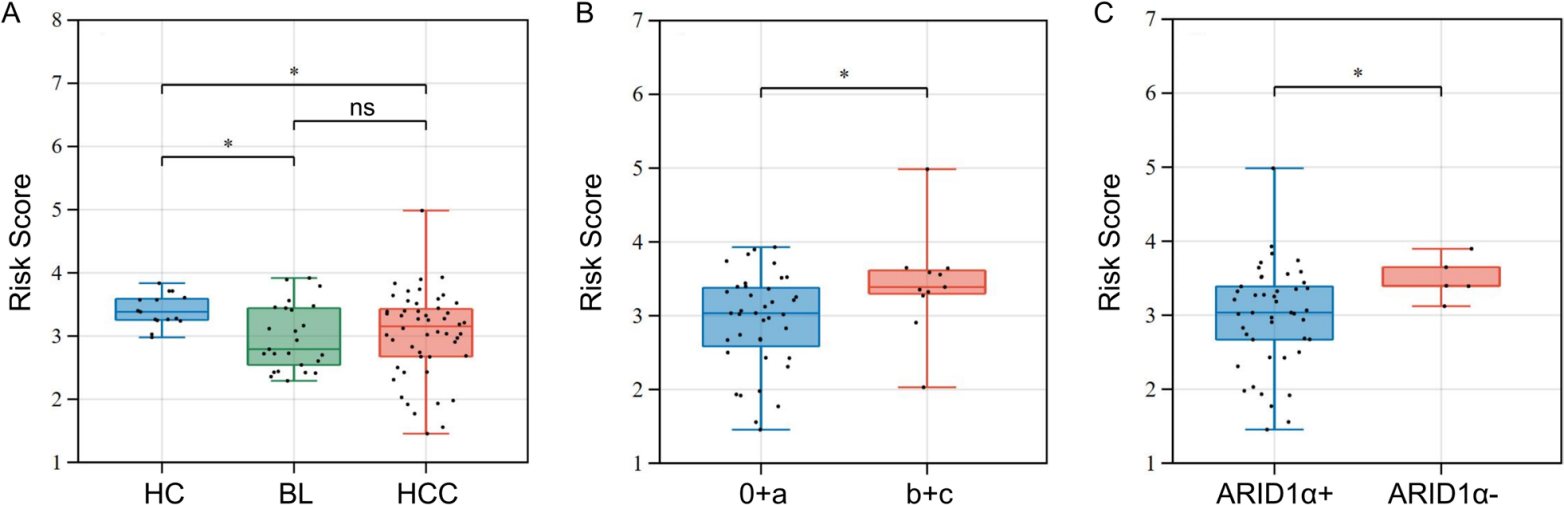
Estimation of risk score by detection of candidate lncRNA using qRT-PCR. Comparison of risk score in different groups (**A**), BCLC stage (**B**) and ARID1α status (**C**). ^*^: *P* < 0.05, ^**^: *P* < 0.01, ^***^: *P* < 0.001, ns: not significant

We compared plasma DDR-related lncRNAs and risk score among different groups. HC showed higher risk score (Fig. 6A, *P* = 0.035), AL031985.3 (Figure S4B, *P* = 0.038) and SNHG4 (Figure S4B, *P* = 0.004) than HCC. However, no differences of risk score, AC012073.1, AL031985.3, AL355574.1, LINC01224 and SNHG4 were found between BL and HCC (Fig. 6B). We further explored the correlations between plasma lncRNAs and clinical features, including Barcelona Clinic Liver Cancer (BCLC) stage and ARID1α status. Patients with advanced BCLC stage had higher plasma AC012073.1 (Figure S4C, *P* = 0.014), AL355574.1 (Figure S4C, *P* = 0.004), LINC01224 (Figure S4C, *P* = 0.004) and SNHG4 (Figure S4C, *P* = 0.016), as well as higher risk score (Fig. 6B, *P* = 0.030). In addition, ARID1α-deficient HCC showed higher plasma AL031985.3 (Figure S4D, *P* = 0.016) and risk score (Fig. 6C, *P* = 0.042). These findings showed that risk score and candidate plasma lncRNAs were related to prognosis.

## Discussion

HCC has a high recurrence rate and is one of the leading causes of tumor-related deaths. The dysfunction of the DDR process has been determined to have important implications for carcinogenesis, progression, treatment and prognosis in HCC. In previous studies, the DDR-related lncRNAs signature for prognostic prediction have been described in many kinds of cancers, such as gastric cancer [15], colon cancer [16], ovarian cancer [17], etc. However, the DDR-related lncRNAs in the HCC prognostic model remains to be clarified.

In this study, we reported a DDR-related lncRNAs signature (including AC012073.1, AL031985.3, AL355574.1, LINC01224 and SNHG4) for prognosis and precise treatment of HCC. Patients with low risk score had significantly longer OS. Risk score was also confirmed to be an independent risk factor for OS in HCC patients. The nomogram consist of TNM stage and risk scores was established, which proved to be a better predictor than the TNM stage alone. Previous studies show that TP53 plays a key role in DDR and more frequently mutated in HCC [18]. Moerover, TP53 mutations have been clinically recognized as an inferior survival indicator for HCC [19]. Our findings showed that TP53 mutation was more frequently mutated in the high-risk group. Significant differences were found in expression of candidate DDR-related lncRNAs in HCC cell lines. We found that patients with advanced BCLC stage had higher plasma AC012073.1, AL355574.1, LINC01224 and SNHG4, as well as higher risk score. In addition, ARID1α-deficient HCC showed higher plasma AL031985.3 and risk score. ARID1α participates in control of the PI3K/AKT/mTOR pathway, immune responsiveness, steroid receptor modulation, DNA damage checkpoints, and regulation of p53 targets and KRAS signaling [20]. More evidence show that ARID1α deficiency is associated with poor prognosis and metastases of HCC [21–23]. These advantages could be helpful to make clinical decisions and make nomogram a superior tool for predicting prognosis.

Increasing evidence suggests that tumor development and progression depend on the complex TME in which they reside, including the tumor cells and their surrounding immune cells [24]. DNA damage response shape both innate and adaptive immune pathways [25]. DDR components enhance cytosolic DNA sensing and its downstream STimulator of INterferon Genes (STING)-dependent signaling, and are involved in the assembly and diversification of antigen receptor genes for lymphocyte development. Moreover, DDR-related sensors and protein complexes can facilitate tumor cell immune evasion [26]. We investigated the infiltration of immune cells in HCC in different risk groups and discovered that the levels of memory B cells, memory-activated CD4 + T cells, follicular helper T cells, M0 macrophages and resting dendritic cells climbed in the high-risk group, and their infiltration abundance increased as the risk score increased. These findings indicate that this risk score can distinguish between immune infiltration characteristics of high- and low-risk groups.

Chemotherapy and immunotherapy are two common therapeutic options for advanced HCC patients. A systematic review reported that immune-related adverse effects (irAEs) could occur in any organ and impact 89% of patients treated with CTLA-4 inhibitors [27]. Early prediction of response for chemotherapy and immunotherapy is essential to improve treatment outcomes and avoid adverse effects. We found that the two risk groups had different sensitivity to different chemotherapy for treating HCC. Interestingly, we found patients with high-risk score were more sensitivity to gefitinib. A recent study suggests that lenvatinib in combination with gefitinib might be a promising strategy to improve clinical outcomes for some HCC patients [28]. Our findings indicated that patients with high-risk score were more sensitive to gefitinib, which could be a a promising target in HCC. In addition, we observed that patients with high-risk score had higher expression of negative immune checkpoints, which indicated that the signature has a potential predictive significance for the efficacy of immunotherapy. Briefly, our findings showed that the DDR-related lncRNAs signature may predict the response of chemotherapy and immunotherapy and identify patients potentially benefiting from the therapy precisely.

However, there are some limitations in our study. Firstly, external validation was missing for lack of expression profiles of lncRNAs and OS data in other databases. Secondly, although the expression levels of the DDR-related lncRNAs were validated by qRT-PCR in cell lines and plasma samples routinely collected in clinical, larger sample sizes were required to make the evidence more solid. Thirdly, the mechanism of how these lncRNAs affect DDR pathway remains unknown. Further research on the relationship between these lncRNAs and DDR genes is necessary.

## Conclusions

In summary, a novel lncRNAs signature based on DDR has been developed, which has an important potential in HCC prognostic prediction and therapeutic response. Our findings suggested a promising insight into DDR-related lncRNAs in HCC and provided a personalized prediction tool for prognosis and drug response.

### Supplementary Information

**Additional file 1: Supplementary Table 1.** 450 DDR gene list download from MSigDB. **Supplementary Table 2.** 76 DDR-related DEGs. **Supplementary Table 3.** 109 DDR-related lncRNAs with prognostic value.

**Additional file 2: Figure S1.** Evaluation and validation of the utility of the DDR-related lncRNAs signature. **Figure S2.** K-M curve of the high- and low- TMB groups. **Figure S3.** Comparison of the relative percentage of 22 immune cells estimated between the high- and low- risk groups. **Figure S4.** Evaluation of candidate lncRNAs using qRT-PCR.

## Data Availability

The data that support the findings of this study are available in this article.

## Abbreviations

AUCs: Area under the curves
BCLC: Barcelona Clinic Liver Cancer
BL: Benign lesions
CI: Confidence interval
DDR: DNA damage repair
DE-lncRNAs: Differentially expressed lncRNAs
DEGs: Differentially expressed genes
DSB: Double-strand break
FBS: Fetal bovine serum
FDR: Fold change
HC: Healthy control
HCC: Hepatocellular carcinoma
HR: Hazard ratio
IC50: Inhibitory concentration
irAEs: Immune-related adverse effects
K-M: Kaplan-Meier
LASSO: Least absolute shrinkage and selection operator
LIHC: Liver hepatocellular carcinoma
LncRNAs: Long noncoding RNAs
MSigDB: Molecular Signatures database
OS: Overall survival
qRT-PCR: Quantitative real-time PCR
ROC: Receiver operating characteristic
STING: STimulator of INterferon Genes
TCGA: The Cancer Genome Atlas
TMB: Tumor mutational burden
TME: Tumor microenvironment
