# Supplementary material for: Identification of a DNA damage repair-related LncRNA signature for predicting the prognosis and immunotherapy response of hepatocellular carcinoma
Source: BMC Genomics. 2024 Feb 8;25:155. doi: 10.1186/s12864-024-10055-1 (PMC10851502; doi:10.1186/s12864-024-10055-1)
Supplement: Supplementary file 2 — Additional file 2: Figure S1. Evaluation and validation of the utility of the DDR-related lncRNAs signature. Figure S2. K-M curve of the high- and low- TMB groups. Figure S3. Comparison of the relative percentage of 22 immune cells estimated between the high- and low- risk groups. Figure S4. Evaluation of candidate lncRNAs using qRT-PCR. [file 12864_2024_10055_MOESM2_ESM.docx]

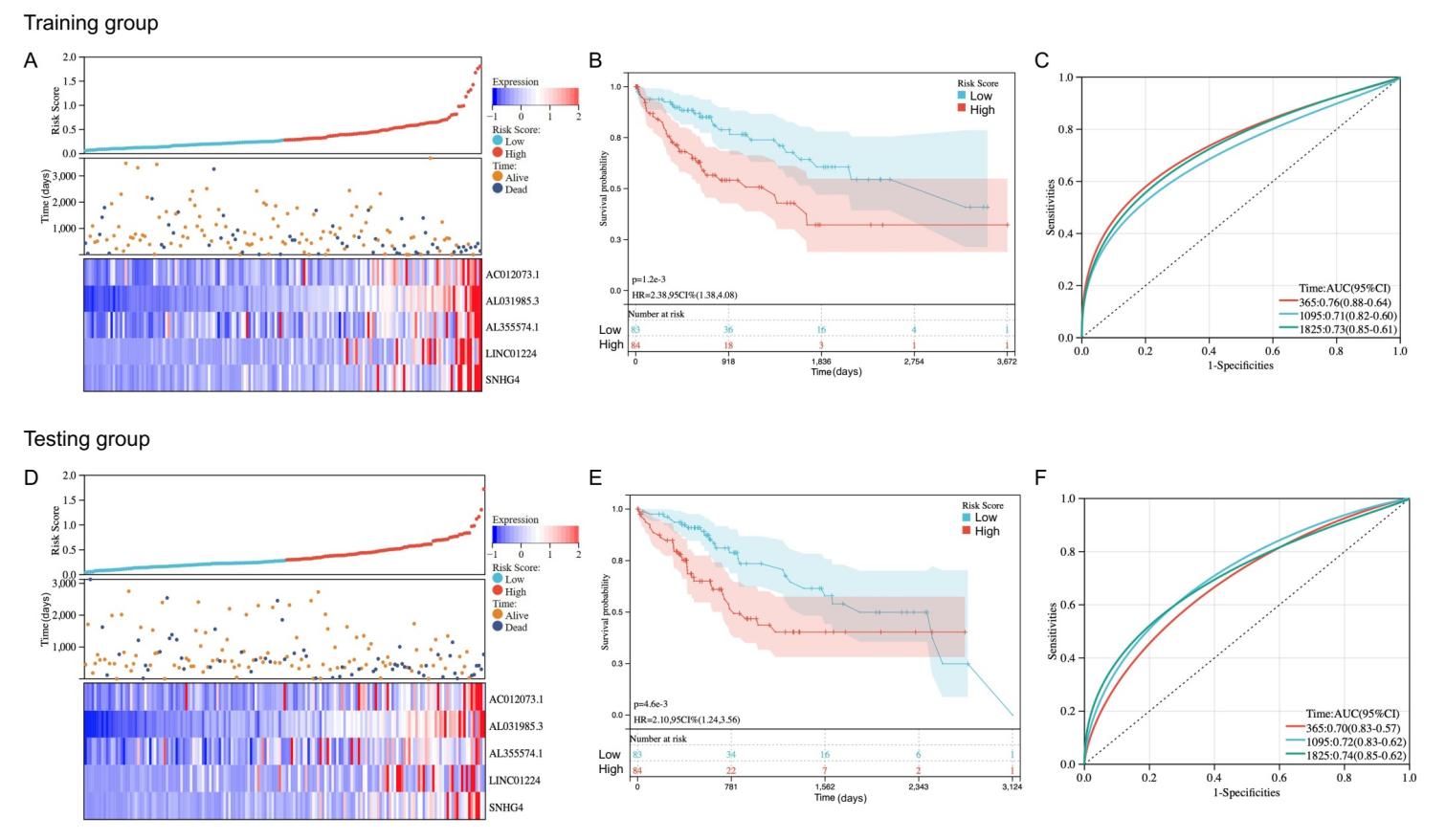


Figure S1. Evaluation and validation of the utility of the DDR-related lncRNAs signature. The distribution of the risk score and scatter plot of survival in the training (A) and testing group (D). K-M curve of the high- and low- risk group in the training (B) and testing group (E). Time-dependent ROC curve for the prognostic prediction of the risk model at 1-, 3-, and 5-year survival time in the training (C) and testing group (F).


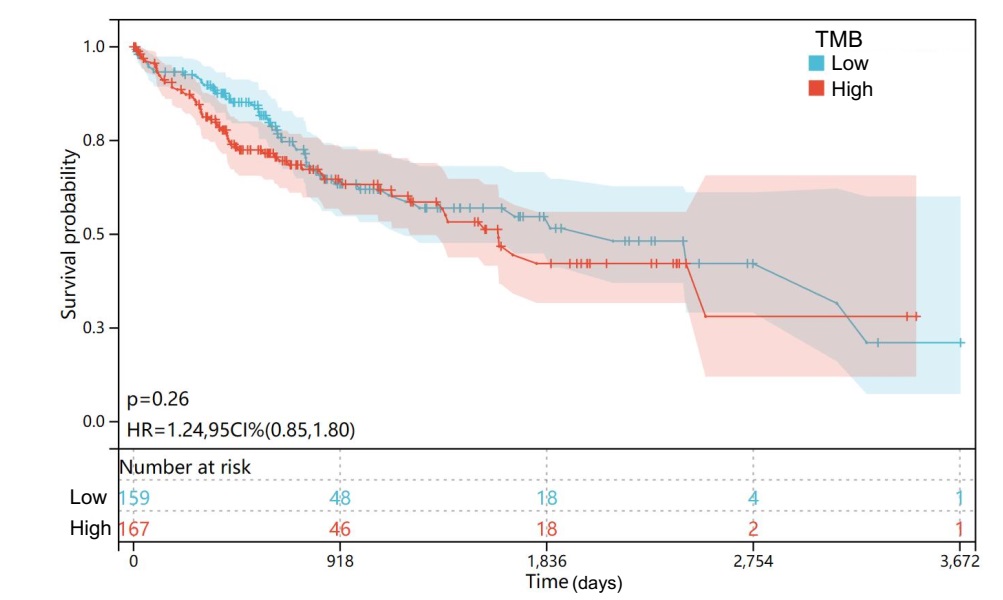


Figure S2. K-M curve of the high- and low- TMB groups.


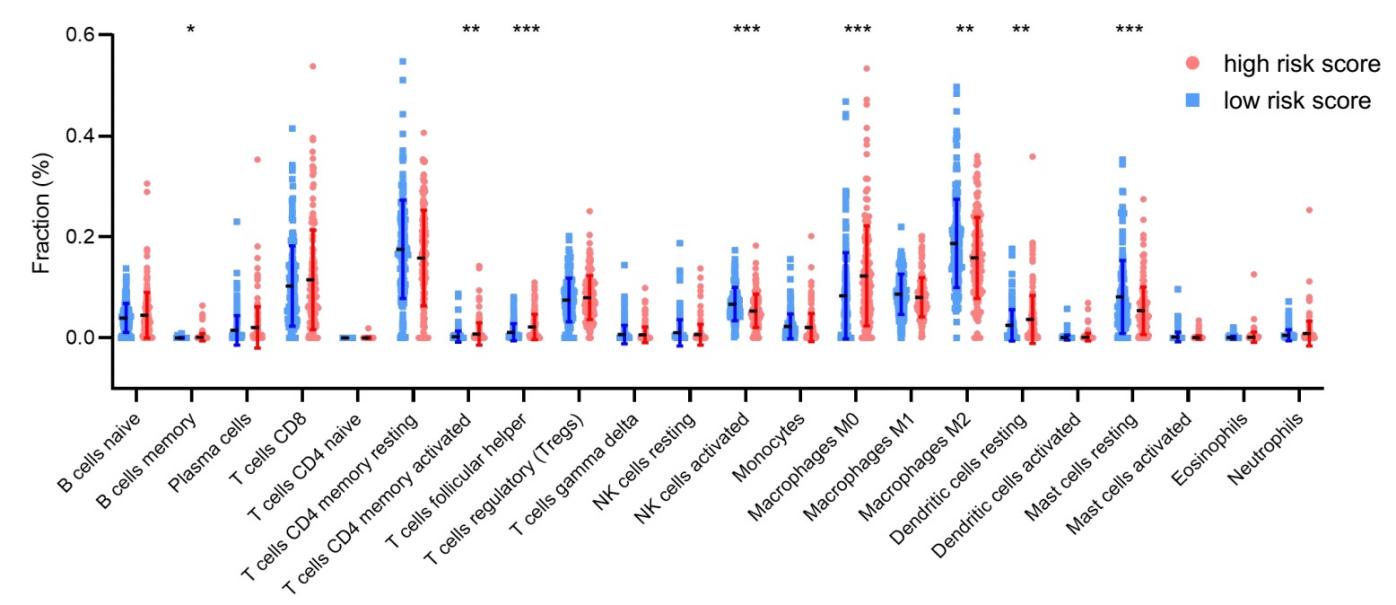


Figure S3. Comparison of the relative percentage of 22 immune cells estimated between the high- and low- risk groups.


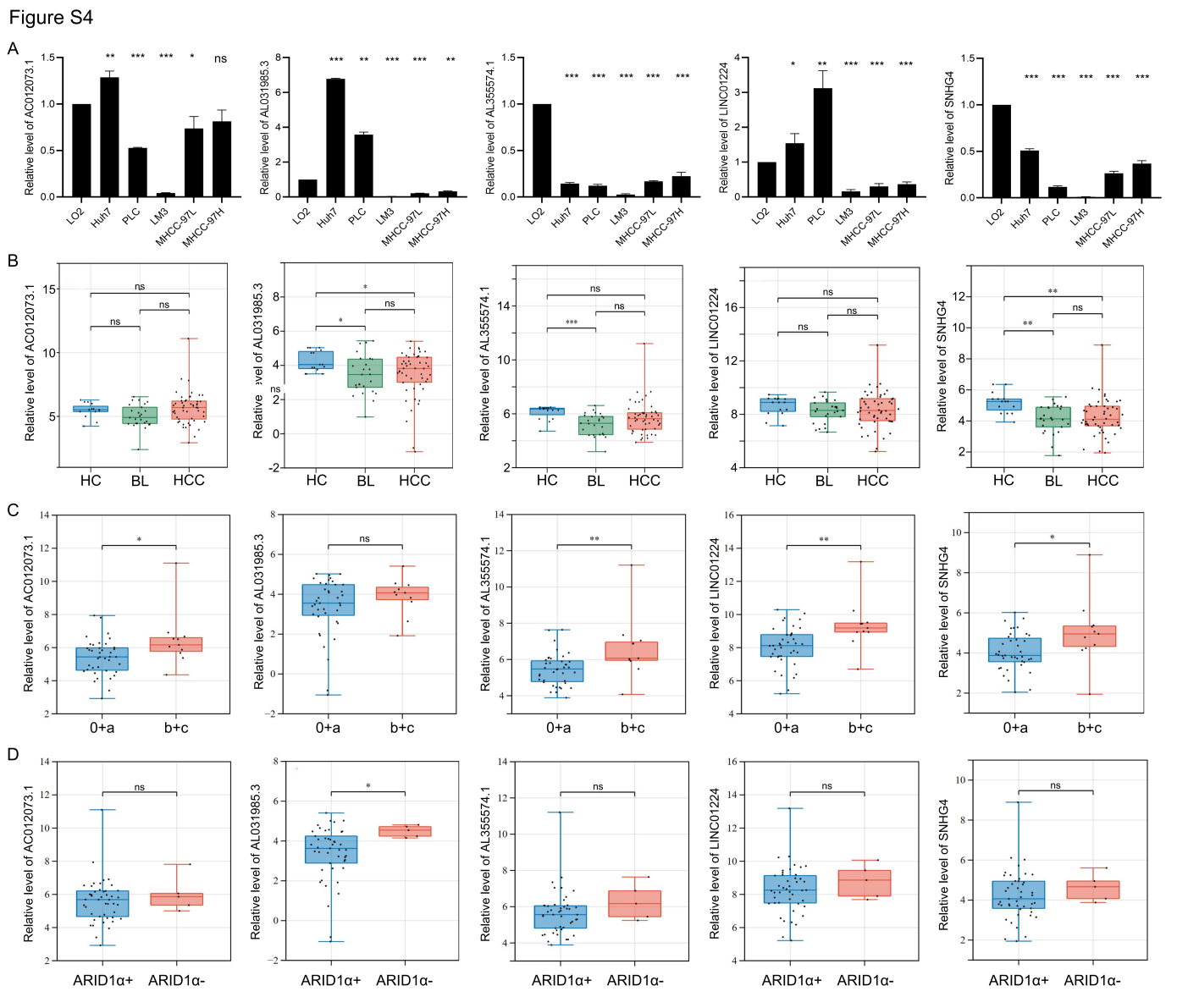


Figure S4. Evaluation of candidate lncRNAs using qRT-PCR. Expression analysis of lncRNAs in various cell lines (A) were normalized to GAPDH. Comparison of candidate lncRNAs in different groups (B), BCLC stage (C) and ARID1α status (D). ^*^: P< 0.05, ^**^: P< 0.01, ^***^: P< 0.001, ns: not significant.
